# Feed Choice Led to Higher Protein Intake in Broiler Chickens Experimentally Infected With *Campylobacter jejuni*

**DOI:** 10.3389/fnut.2018.00079

**Published:** 2018-09-05

**Authors:** Christian Visscher, Linus Klingenberg, Julia Hankel, Ralph Brehm, Marion Langeheine, Ariane Helmbrecht

**Affiliations:** ^1^Institute for Animal Nutrition, University of Veterinary Medicine Hannover, Foundation, Hannover, Germany; ^2^Institute for Anatomy, University of Veterinary Medicine Hannover, Foundation, Hannover, Germany; ^3^Evonik Nutrition & Care GmbH, Hanau, Germany

**Keywords:** broiler, *Campylobacter jejuni*, feed choice, protein requirements, performance

## Abstract

In 2016, *Campylobacter* was the most commonly reported gastrointestinal bacterial pathogen in humans in the European Union with 246,307 reported cases. Of these cases, 83.6% were *Campylobacter jejuni*. The objective of the present study was to investigate to what extent an infection with *C. jejuni* alters the feed intake behavior of broiler chicks in terms of protein intake. This was done to see if, conversely, measures of control could be derived. In total, 300 commercial broilers of the Ross 308 line were allocated to four different groups, including five replications of 15 chickens each. In two groups, a conventional diet [216 g CP/kg dry matter (DM)] and in the two choice diet groups, diets with different levels of crude protein (286 and 109 g CP/kg DM, respectively) were fed between day 14 and day 42. An intake of both choice diets at a ratio of 3:2 resulted in a composition of consumed feed identical to that of the control concerning composition, energy and nutrient content. One group of each feeding concept was infected artificially with *C. jejuni* at day 21 by applying an oral *C. jejuni*-suspension containing 5.26 ± 0.08 log_10_ colony forming units of *C. jejuni* to three out of 15 chickens. No significant differences concerning *C. jejuni* prevalence and excretion could be seen. Broilers infected with *C. jejuni* chose a higher amount of the high protein choice diet in comparison to *C. jejuni* negative broilers. This resulted in a significantly (*p* < 0.0001) higher content of crude protein in the consumed diet (198 ± 3.09 g CP/kg DM and 208 ± 8.57 g CP/kg DM, respectively). Due to *C. jejuni* infection, a significant increase in crude mucin in excreta at day 42 was seen in experimentally infected groups (62.6 ± 4.62 g/kg DM vs. 59.6 ± 6.21 g/kg DM, respectively; *p* = 0.0396). There were significantly deeper crypts in infected birds (256 ± 71.6 vs. 234 ± 61.3 μm). In summary, *C. jejuni* infections significantly alter the feed intake behavior of broiler chickens regarding higher protein intake. Therefore, targeted manipulation of protein supply could be tested for limiting the spread of infection.

## Introduction

*Campylobacteriosis* is one of the world's most important diarrheal diseases (1). In 2016, *Campylobacter* was the most commonly reported gastrointestinal bacterial pathogen in humans in the European Union (EU) and has been the case since 2005 (2). The number of reported confirmed cases of human *campylobacteriosis* was 246,307, with an EU notification rate of 66.3 per 100,000 population (2). This represented an increase of 6.1% compared with 2015 (2). Of confirmed cases reported in the EU, 83.6% were *Campylobacter jejuni* [*C. jejuni*; (2)].

Carbohydrates are only a minor substrate for the metabolism of *Campylobacter*. They are used only to a very limited extent (3). This is different than most other intestinal bacteria. The metabolism of *C. jejuni* relies on utilizing amino acids (3). Serine, aspartate, glutamate and proline are the preferred amino acids (4, 5). These amino acids are essential for the formation of the mucin-glycoproteins of the intestinal mucus layer (6, 7). Thus, they are most frequently included in both, the mucus layer and the excreta of poultry (8). In principle, threonine is of structural importance in the mucin protein backbone (9). The main constituents of the intestinal mucus layer are the mucins, which are produced by the goblet cells (10, 11). *C. jejuni* possesses very successful strategies to invade and colonize the mucus layer (12). The presence of mucins appears to be essential for the survival and growth of *C. jejuni* (12, 13). The crude protein content in the diet is relevant for mucin synthesis (14). Therefore, the crude protein supply is in parts responsible for the thickness and composition of the intestinal mucus layer (14, 15). Reduced crude protein content leads to reduced mucin production as well as a decrease in its release into the intestinal tract of broilers (14). In the context of enteral infections, there is an increased production of mucins associated with high amino acid demand (7, 16, 17). In addition to this demand for amino acids, the immune cells themselves assume high glucose consumption for immunological mechanisms (18). The mobilization of fat deposits provides energy reserves (19, 20), while metabolites of amino acid breakdowns can be used for enhanced gluconeogenesis (18, 20). This high variability in the demand for amino acids also affects the feed intake behavior of animals (21–24). There is much to be said for the homeostatic regulation of amino acid uptake, which is based on an extremely complex but not yet well-researched interplay of different mechanisms (24). In addition to this ability to detect an unbalanced amino acid composition of the diet, broilers can find out the component that best suits their needs (22, 25–27). In empirical scientific work, this ability is used to investigate the influence of different external and internal factors on the feed intake behavior of the animals in feeding experiments (election experiments) with a choice between different animal feeds (26, 28, 29). However, it should be noted that in feeding trials dealing with the level of protein intake that feed intake essentially depends on the energy content of the diet, whereby the total feed intake is negatively correlated with the energy content (26). In growing poultry, the protein requirement remains relatively constant, while the energy requirement increases to some degree (22). Broilers tend to meet their energy needs by minimizing total feed intake (22).

The hypothesis in the present experiment was that broilers show different dietary intake behavior depending on *C. jejuni* infection. Therefore, targeted manipulation of the protein supply and amino acid pattern could be tested for limiting the spread of infection in further studies.

## Materials and methods

The aim of the investigations was to analyze the influence of an experimental *C. jejuni* infection in broilers on feed intake behavior. In control groups, a compound feed regime (SP-diet) was used. In the choice experiment, birds were given the option to select between a low protein diet (CD^CP−^-diet) and a high protein diet (CD^CP+^-diet). At the same time, potential effects of the experimental *C. jejuni* on the spread of infection, the level of *C. jejuni* excretion, performance of birds, histology of the ileum and the mucin content in excreta were of interest. The artificial infection was done in a seeder model. Therefore, animal experiments were performed in accordance with the German rules and regulations and approved by the Ethics Committee of Lower Saxony for Care and Use of Laboratory Animals, LAVES (Niedersächsisches Landesamt für Verbraucherschutz und Lebensmittelsicherheit; reference: 33.19-42502-05-15A540).

### Animals and housing

Day of hatching chicks (day 0 = d 0) of both sexes (*N* = 300; ROSS 308) were obtained from a commercial hatchery (BWE-Brüterei Weser Ems, PHW Gruppe/LOHMANN & Co. AG, Visbek, Germany). For the first 14 days, the birds were housed in four identical floor pens littered with wood shavings. The temperature profile in the pen started with a temperature of about 34–36°C. During the trial, the temperature was lowered by about 1°C every 2 days, reaching a minimum temperature of about 20°C. The photoperiod beginning from d 4 was 16 h of light and 8 h of darkness during the whole trial which complies with the local regulations on keeping chickens (Tierschutz-Nutztierhaltungsverordnung), which provides for 6 h of dark time plus dimming periods of light.

After a fourteen-day rearing phase, the animals were transferred in-house to the experimental unit (security level 2, this means standard biosecurity and institutional safety procedures). Following this, the animals were randomly subdivided into 20 subgroups in a 2 × 2 factorial design with two different diets (SD-diet, CD-diet consisting of the components CD^CP+^ and CD^CP−^) and a different infection modus (CN, *Campylobacter* Negative, CP, *Campylobacter* Positive) and the following combinations thereof (SDCN-Standard Diet, *Campylobacter* Negative; CDCN-Choice Diet, *Campylobacter* Negative; SDCP- Standard Diet, *Campylobacter* Positive; CDCP-Choice Diet, *Campylobacter* Positive). All birds in the study were individually tagged with wing-tags.

**Figure 1:**
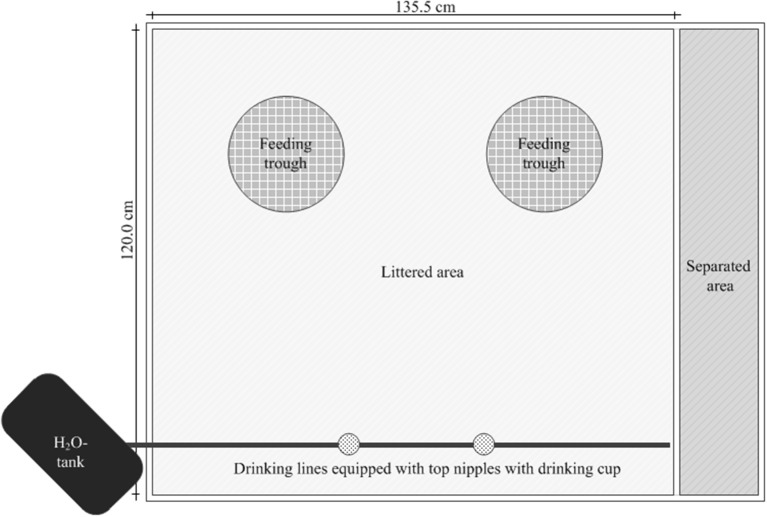
Presentation of the arrangement of the feeding troughs and the water lines for control and experimental groups in a box.

The animals were kept in modified boxes (AviMax, Big Dutchman AG, Vechta, Germany) with solid flooring littered with wood shavings (1 kg/m^2^) in groups of 15 animals (“subgroup”). Each subgroup had an unrestricted available floor space (total area minus the area under the trough) of 1.45 m^2^. Rearing took place up to the end of the 4-week experimental period (beginning at d 42) or rather up to dissection (d 43–45; Figure 1).

### Feeding regime and feed analysis

In the rearing phase (d 0–13) a conventional pelleted starter diet (starter) was fed for 1 week, followed by a subsequent 7-day phase with a commercially available pelleted grower diet (grower; Best 3 Geflügelernährung GmbH, Twistringen, Germany). Diets for the main experimental period (d 14 onwards) were produced in cooperation with Evonik Nutrition & Care GmbH (Hanau-Wolfgang, Germany). A manufacturer for trial diets (Research Diet Services BV, Wijk bij Duurstede, the Netherlands; Table 1) produced the different diets. The standard diet (SD-diet) was designed in accordance with a commercially available standard fattening diet concerning ingredients and composition (Tables 1–3). The energy and nutrient content to a certain amount exceeded the recommendations for the energy and nutrient supply of the laying hens and fowls (broilers) of the Committee for Needs Standards of the Society for Nutritional Physiology (30). The other two groups were given the opportunity to freely choose between two different diets. This choice diet consisted of a high protein component (CD^CP+^-diet: 286 CP/kg DM) and a low protein component (CD^CP−^-diet: 109 g CP/kg DM). These two compound feeds were based on the SD-diet, modified only in their content of wheat and soybean meal. Due to the specifically formulated composition of the two components of the CD-diet (CD^CP+^-diet, CD^CP−^-diet; Table 1), a bird consuming three parts CD^CP+^-diet (60%) and two parts CD^CP−^-diet (40%) ingested a feed mixture that referred to an identical botanically and chemically to the standard feed (SD-diet). All diets were offered *ad libitum*. Circular feeding troughs, two for each box, were used during the entire trial (Crown Poultry Feeders, New Zealand). In the control groups (SDCN, SDCP), both troughs contained an identical diet. In the experimental groups (CDCN, CDCP), each trough contained one of the two CD-diets (CD^CP+^-diet, CD^CP−^diet). Water was offered *ad libitum* in double-cylinder plastic bell drinkers in the rearing phase, later via drinking lines equipped with Top Nipples with a drinking cup (Big Dutchman International GmbH, Vechta-Calveslage, Germany). The water was treated with chlorine-oxygen preparation at a concentration of 0.3 mg/L to kill any *C. jejuni* in the drinking water (Virbac Clean Pipe, VIRBAC Tierarzneimittel GmbH, Bad Oldesloe, Germany). Water and feed samples were tested for *C. jejuni*. Both substrates were originally *C. jejuni* negative.

**Table 1:** Ingredient composition of the standard protein diet and high protein diet and low protein diet of the choice feeding concept for the main experimental period (days 21–42).

**Ingredients (in %)**	**SP-diet^a^**	**CD^CP+^-diet**	**CD^CP-^-diet**	**CD^CP+^/CD^CP-^-diet [60/40]**
Wheat	41.4	25.3	65.5	41.4
Corn	25.0	25.0	25.0	25.0
Soybean meal^b^	24.1	40.2	0.00	24.1
Soybean oil	5.16	5.16	5.16	5.16
Monocalciumphosphate	1.37	1.37	1.37	1.37
Calcium carbonate	1.28	1.28	1.28	1.28
Premix “Blank Poultry”^c^	0.50	0.50	0.50	0.50
L-Lysin-HCl^®^^d^	0.26	0.26	0.26	0.26
Sodium bicarbonate	0.25	0.25	0.25	0.25
MetAMINO^®^^e^	0.25	0.25	0.25	0.25
Sodium Chloride	0.20	0.20	0.20	0.20
ThreAMINO^®^^f^	0.10	0.10	0.10	0.10
L-Isoleucin	0.06	0.06	0.06	0.06
ValAMINO^®^^g^	0.06	0.06	0.06	0.06

*SP-diet, Standard protein diet; CD^CP+^-diet, High protein choice diet; CD^CP−^diet, Low protein choice diet; CD^CP+^/CD^CP−^-diet [60/40], Composition of the choice diet with 60% CD^CP+^-diet and 40% CD^CP−^diet*.

a *The sum of all the ingredients does not equal 100 due to rounding differences*.

b *48% crude protein*.

c *Carrier: cornflour; content per kg: iron (16,000 mg), copper (2,400 mg), manganese (17,000 mg), zinc (12,000 mg), iodine (160 mg), selenium (30 mg), vitamin A (2,000,000 IU), vitamin D3 (500,000 IU), vitamin E (10,000 mg), vitamin K3 (300 mg), vitamin B1 (400 mg), vitamin B2 (1,500 mg), vitamin B6 (700 mg), vitamin B12 (4,000 μg), niacin (7,000 mg), D-pantothenic acid (2,400 mg), choline chloride (92,000 mg), folic acid (200 mg), biotin [40 mg]*.

d *78.0% L-Lysine*.

e *99.0% DL-Methionine*.

f *98.5% L-Threonine*.

g *98.0% L-Valine*.

**Table 2:** Concentrations of ingredients and energy content after chemical analysis in the standard protein diet and in the high protein diet and low protein diet of the choice feeding in the experimental period (days 21–42).

**Item**	**SP-diet^1^**	**CD^CP+^-diet**	**CD^CP-^-diet**	**CD^CP+^/CD^CP-^-diet [60/40]**
Dry matter [g/kg diet]	883	888	877	884
Crude ash [g/kg DM]	53.0	60.9	41.1	53.0
Crude fat	80.6	76.5	78.4	77.2
Crude fiber	28.9	28.9	25.1	27.4
Crude protein	216	286	109	215
Nitrogen free extract^a^	621	547	747	627
Starch	460	366	618	467
Sugar	46.1	56.8	27.5	45.1
Calcium	9.42	9.93	9.39	9.71
Phosphorus	7.78	8.37	6.83	7.75
Potassium	8.32	12.3	4.11	9.00
Sodium	1.86	1.77	1.98	1.85
Chloride	2.74	2.61	2.88	2.72
Magnesium	1.85	2.27	1.22	1.85
Sulfur	2.96	3.47	2.25	2.98
Metabolisable energy AME_N_^b^ [MJ/kg DM]	14.4	13.9	15.1	14.4

SP-diet, Standard protein diet; CD^CP+^-diet, High protein choice diet; CD^CP−^-diet, Low protein choice diet; CD^CP+^/CD^CP−^-diet [60/40], Composition of the choice diet with 60% CD^CP+^-diet and 40% CD^CP−^-diet

a *Nitrogen-free extract, DM - (crude ash + crude fat + crude fiber + crude protein)*.

b *Metabolisable energy AME_N_ (MJ/kg DM), 0.1551×% crude protein+0.3431×% crude fat+0.1669×% starch+0.1301×% sugar*.

**Table 3:** Amino acid content in the standard protein diet as well as in the high protein diet and low protein diet of the choice feeding in the experimental period (days 21–42).

**Item**	**SP-diet**	**CD^CP+^-diet**	**CD^CP-^-diet**	**CD^CP+^/CD^CP-^-diet [60/40]**
Arginine [g/kg DM]	13.8	19.7	4.72	13.7
Cysteine	4.55	5.12	3.17	4.34
Isoleucine	9.39	13.1	3.96	9.42
Leucine	16.2	21.9	7.72	16.2
Lysine	12.8	18.0	5.37	13.0
Methionine	6.10	6.84	4.90	6.06
Phenylalanine	10.4	14.0	4.68	10.3
Threonine	8.02	11.8	4.38	8.85
Valine	10.3	14.1	4.93	10.4
Alanine	9.18	12.4	4.33	9.17
Aspartic acid	19.9	28.4	5.43	19.2
Glutamic acid	41.2	53.0	24.6	41.7
Glycine	8.56	11.7	4.00	8.63
Histidine	5.44	7.58	2.51	5.55
Proline	13.9	17.7	9.40	14.4
Serine	10.2	14.5	4.80	10.6
Tyrosine	8.02	9.88	3.28	7.24

*SP-diet, Standard protein diet; CD^CP+^-diet, High protein choice diet; CD^CP−^diet, Low protein choice diet; CD^CP+^/CD^CP−^-diet [60/40], Composition of the choice diet with 60% CD^CP+^-diet and 40% CD^CP−^-diet*.

Diets were analyzed by standard procedures in accordance with the official methods of the VDLUFA (31). The dry matter content (DM) was determined by drying to the weight constancy at 103°C, whereas the raw ash was analyzed by means of incineration in the muffle furnace at 600°C for 6 h. The total nitrogen content was determined in accordance with the DUMAS combustion method by means of the analyzer Vario Max® (Elementar, Hanau, Germany). The crude fat content was determined in the soxleth apparatus and the content of crude fiber was analyzed after washing in dilute acids and alkalis. Starch determination was carried out polarimetrically (Polatronic E, Schmidt und Haensch GmbH & Co., Berlin, Germany). The sugar content was analyzed by the method in accordance with Luff-Schoorl by titration, whereas the mineral content was determined by atomic absorption spectrometry (Unicam Solaar 116, Thermo, Dreieich, Germany). Amino acids were determined by ion-exchange chromatography (AA analyser LC 3000, Biotronic, Maintal, Germany).

### Experimental infection and sampling

Prior to *Campylobacter* challenge, there was a two-step procedure to ensure that there was no *Campylobacter* colonization prior to experimental infection. This was done at d 18 in five animals per subgroup. Three days later, at d 21, this was performed for each individual animal (*N* = 300). Samples were taken by means of a cloacal swab (Cary Blair smear test system, Süsse Labortechnik GmbH & Co. KG, Gudensberg, Germany).

At d 21, in each subgroup, three of 15 broilers in a box (*n* = 5 for groups SDCP and CDCP each) were administered orally with a *C. jejuni* suspension. A field strain of *C. jejuni* was used for experimental infection (32). This isolate had been identified as *C. jejuni* both culturally and mass-spectrometrically (MALDI-TOF MS; AniCon Labor GmbH, Höltinghausen, Germany). Preparation of the conserved strain for experimental infection was done as previously described (33). The infection strain was used in its stationary growth phase (24–48 h) for preparing the inoculum. *C. jejuni* was resuspended in isotonic 0.9% sodium chloride solution (~10,000 CFU/2 mL). Bacterial suspension for infection was administered orally in three out of 15 randomly selected animals. A button cannula (single-button cannula, sterile, 1.0 × 100 mm, Meiser Medical GmbH, Neuenstein, Germany) was used for application. Analogous to the groups with experimental infection, three of 15 animals were randomly selected from the non-infected control groups (SDCN and CDCN). These animals were administered 2 mL of a sterile sodium chloride solution by means of a button cannula.

At days 22, 23, 24, 25, 28, 35, and 42, individual samples in groups SDCP and CDCP were taken in an identical manner (*n* = 75/group) as described above. In the groups SDCN and CDCN, regular spot-checks of five randomly selected animals per subgroup were examined for *C. jejuni* occurrence. Twenty-one days following experimental infection (beginning at d 42), animals from all groups were qualitatively tested concerning their *Campylobacter* status in accordance with DIN EN ISO 10272-1:2006 (see paragraph “Bacterological Analyses”). Quantitative analyses by determining the colony-forming units of *C. jejuni* in excreta of seeder birds in groups SDCP und CDCP were performed at days 23, 32, and 38. For excreta collection, the corresponding animals were individually placed in purified, disinfected 10 L plastic bucket (26.5 cm) in order to collect freshly dropped excreta.

For determining the mucin content samples in fresh excreta (*n* = 3 pooled samples per box) of the birds, samples were collected from each box at d 20 and d 42 in accordance with established methods (34). Within the infection trial, sampling was omitted to minimize the risk of transmitting infection between boxes. The collected excreta were then removed from each box, thoroughly mixed, and processed in parts for further analysis (mucin content).

Dissection of the animals was performed by standard protocol approved by the Animal Care Committee on three consecutive days (d 43, 44, and 45). The contents of the two ceca were removed under sterile conditions and placed in a screw cup (screw cup 100 mL, PP, Sarstedt AG & Co., Nümbrecht, Germany) for all animals in groups SDCP and CDCP.

### Bacteriological analyses

Bacteriological examination at bird level (qualitative analyses) was based on DIN EN ISO 10272-1:2006, taken from the official collection of analysis methods in accordance with § 64 LFBG. The sample matrix was incubated in a one-to-nine ratio (sample:Bolton-broth) in sterile 5 mL tubes mounted with a vent cap (Sarstedt AG & Co., Nümbrecht, Germany). Incubation lasted 4 h at 37°C, followed by 44 ± 4 h at 41.5°C under a microaerobic atmosphere (oxygen content of 5 ± 2%, carbon dioxide content of 10 ± 3%; DIN EN ISO 10272-1:2006). Enrichment in Bolton-broth was followed by streaking samples onto two solid selective culture media (mCCD agar and Karmali agar; Oxoid Germany GmbH, Wesel, Germany) with sterile inoculation loops. Selective cultures were incubated again for 44 ± 4 h at 41.5°C in a microaerophilic atmosphere. Presence of *Campylobacter* was confirmed by analyzing individual colonies. This was done by phase contrast microscopy (Distelkamp-Electronic, Kaiserslautern, Germany) and biochemical methods (apiCampy, bioMérieux SA, Marcy-LÈtoile, France).

Quantitative bacteriological examination was done by a ten-fold dilution series (0.5 g sample material in 4.5 mL of sterile Phosphate Buffered Saline—PBS) with PBS (Phosphate Buffered Saline, Oxoid Germany GmbH, Wesel, Germany). In duplicate, 100 μL of each dilution was plated onto mCCD agar (Oxoid Germany GmbH, Wesel, Germany). After incubation (microaerophilic atmosphere: 44 ± 4 h at 41.5°C), the colonies were counted and an average value from the two duplicate experiments was taken for calculating the CFU/g intestinal content. In accordance with DIN EN ISO 10272-2:2006, only plates with more than 30 and fewer than 300 colonies were considered.

### Analysis of mucin content and histological investigations

The content of total mucin was determined in birds‘ pooled excreta. Quantifying the water-soluble and ethanol-precipitable fraction of excreta was carried out in accordance with modified methods (9, 35) as described by Visscher et al. (33).

For histological investigations, an ~1 cm long piece was removed from the apex of the right cecum, ~1 cm proximal to the apex, and fixed in 4% formaldehyde for 48 h. After fixation, tissue samples were embedded in paraffin using standard techniques (36). For histological evaluation, 4 μm sections of all samples were stained with HE using established protocols (36). For determining the number of goblet cells in the cecal crypts, sections were viewed with a Zeiss axioscope (Carl Zeiss Jena GmbH, Jena, Germany). Sample analysis was done in accordance with established methods (9) with slight modifications. The depth of five complete vertically oriented crypts was measured in each of the blinded samples. The number of goblet cells of the crypt was counted. The number of goblet cells of the individual, measured crypts of each sample was converted to a standard crypt depth of about 250 μm for comparison between groups.

### Performance parameters

The body weight of the birds was recorded individually at the beginning of days 7, 14, 21, and 42 (PCE TB 30, PCE Instruments, Meschede, Germany). The animal losses were taken into account for all analyzed parameters. The feed and water intake were recorded at the level of the box (20 boxes in total; *n* = 5 subgroups/boxes per group). The feed conversion ratio (FCR) reflected feed consumption per kilogram of body weight gain. Protein efficiency was calculated as the increase in body weight per kilogram of crude protein intake. The feed-water ratio was calculated as the ratio of water intake to feed intake. At the time of dissection (d 43, 44, and 45), the slaughtering weight (body with feathers and without head, feet, gastrointestinal tract, liver, gallbladder, spleen, heart) and the slaughtering exploitations were calculated as a quotient of slaughter weight to the body mass (in %).

### Statistical analyses

The statistical analysis of the collected data was performed using the Statistical Analysis System for Windows the SAS® Enterprise Guide®, version 9.3 (SAS Institute Inc., Cary, USA). The normal distribution of the residues was tested first with a Shapiro–Wilk test before comparing mean values. Normally distributed data were examined in parts for differences in means by a two-factorial analysis of variance with “diet” (SD-diet, CD-diet) and “infection” (CN, CP) as independent variables as well as multiple pairwise comparisons between combinations of variables (Fisher's smallest significant difference). Due to the study design, the factors “diet” and “infection” were not always to be classified as classic independent factors. The SD-Diet was fixed for energy and nutrient composition. Therefore, the effect of the infection on the parameters related to the choice of feed was not done by a two-factorial evaluation because the animals in the SDCP subgroup had no choice. Here the focus was then placed on the comparison between combined factors. Furthermore, half of the subgroups were not experimentally infected with *C. jejuni*. Therefore, a two factorial analysis of the quantitative excretion of the bacteria was not necessary.We sometimes were forced to make pairwise comparison of the fixed combination of CD groups (CDCN, CDCP) in comparison to the fixed parameters from the SD groups. Non-normalized data were processed with a Wilcoxon signed-rank test in pairs to investigate differences in the mean values. For comparing a sample with a constant named above or for comparisons according to *C. jejuni*, a one-sample *t*-test was used for normal distributed data. For uniform distribution of the sample, two-dimensional frequency distributions of categorial features were checked for dependency with the Pearson‘s Chi square homogeneity test. Otherwise, the Fisher's exact test was used. Correlation analyses were carried out on normal distributed data using Pearson's correlation coefficient. Non-normalized data were analyzed using Spearman's rank correlation coefficient. At *p* < 0.05, differences in the mean values, dependence of the frequency distribution or a correlation were regarded as significant.

## Results

The experiment ran completely without complications. Mortality was 1.33%. Three out of 300 broilers used in the experiment died during the experiment, one had to be euthanized (losses in %: SD-diet: 1.33%, CD-diet: 1.33%; CN: 0.67%; CP: 2.00%; SDCN: 1.33%, CDCN: 0.00%, SDCP: 1.33%, CDCP: 2.66%).

### *campylobacter* excretion

Before and at the time of the experimental infection, all animals in the experiment were *Camplylobacter* spp. negative in cloacal swab samples (*N* = 300).

Experimental infection in groups SDCP and CDCP took place at d 21. The inoculum for the experimental infection contained an average of 5.26 ± 0.08 log_10_ CFU *C. jejuni* per bird (2 mL). Already 1 day after this infection, an excretion of *C. jejuni* could be seen with cultural techniques (Table 4). Seven days after experimental infection in both groups, 100% of animals were *C. jejuni* positive in excreta. During the trial, there were no significant differences between groups.

**Table 4:** Prevalence of *C. jejuni* in cloacal swabs of all animals and counts of *C. jejuni* in the excreta of the seeder birds (*n* = 15 per group) at days 23, 32, and 38 as well as total counts of *C. jejuni* in the caecal content of all animals on the day of dissection.

	**Diet**				
	**SP-diet**		**CD**^**CP+**^**/CD**^**CP-**^**-diet**		***P*-value**
**Item**	**% positive**	**n (pos./total)**	**% positive**	**n (pos./total)**	
**PREVALENCE ON GROUP LEVEL**					
Day 18	0.00	(0/25)	0.00	(0/25)	1.0000
Day 21	0.00	(0/75)	0.00	(0/75)	1.0000
Day 22	1.33	(1/75)	8.00	(6/75)	0.1162
Day 23	18.7	(14/75)	17.3	(13/75)	0.8317
Day 24	38.7	(29/75)	42.7	(32/75)	0.6180
Day 25	70.7	(53/75)	77.3	(58/75)	0.3520
Day 28	100	(75/75)	100	(75/75)	1.0000
Day 35	100	(75/75)	100	(75/75)	1.0000
Day 39	100	(75/75)	100	(73/73)	1.0000
Day 42	100.0^a^	(75/75)	100.0^a^	(73/73)	1.0000
**QUANTITATIVE COUNTS *C. jejuni* Excreta Seeder Birds (log_10_ cfu/g)**					
	Mean	SD	Mean	SD	P-value
Day 23	4.11	1.44	3.75	2.12	0.5906
Day 32	5.00	0.75	5.06	0.79	0.8551
Day 38	4.15	0.82	3.58	1.55	0.2454
**QUANTITATIVE COUNTS *C. jejuni* Caecal Content (log_10_ cfu/g)**					
Dissection	7.02	0.85	6.87	1.12	0.6878

*SP-diet, Standard protein diet; CD^CP+^-diet, High protein choice diet; CD^CP−^diet, Low protein choice diet; CD^CP+^/CD^CP−^-diet, Choice diet*;

a,b *Values within a row with different superscripts differ significantly at p < 0.05*.

The mean values of log_10_ CFU *C. jejuni* in the excreta of the seeder animals (15 animals per group, three animals per subgroup) as well as the mean counts of *C. jejuni* in the cecal content did not differ between the SDCP and CDCP groups (Table 4). The SDCN and CDCN groups remained *C. jejuni* negative up to the end of the trial.

### Feed intake and performance parameters

The SD-diet groups were given a diet with an energy content of 14.4 MJ ME/ kg DM throughout the experimental phase. The energy density in the recorded CD-diets of the experimental groups showed maximum deviation of 0.17 MJ ME/ kg DM (+1.18%) in the experimental group CDCN between d 14 and d 21. By comparison, the CP-content in the ingested diet of the experimental group CDCN was 13% lower than the CP-content of the SD diet during the same period (Table 5).

**Table 5:** Energy and crude protein concentration in diets as well as feed and nutrient intake.

**Item**	**CN**				**CP**			
	**SP-diet**		**CD**^**CP+**^**/CD**^**CP-**^**-diet**		**SP-diet**		**CD**^**CP+**^**/CD**^**CP-**^**-diet**	
	**Mean**	**SD**	**Mean**	**SD**	**Mean**	**SD**	**Mean**	**SD**
**ME-CONTENT [MJ ME/kg DM]**								
Day 14–20	14.4^b^	0.00	14.6^a^	0.07	14.4^b^	0.00	14.5^a^	0.05
Day 21–27	14.4^a^	0.00	14.4^a^	0.03	14.4^a^	0.00	14.4^a^	0.07
Day 28–34	14.4^b^	0.00	14.5^a^	0.05	14.4^b^	0.00	14.4^b^	0.05
Day 35–41	14.4^b^	0.00	14.6^a^	0.06	14.4^b^	0.00	14.5^ab^	0.09
Day 21–41	14.4^b^	0.00	14.5^a^	0.02	14.4^b^	0.00	14.4^b^	0.06
**CP-CONTENT [g/kg DM]**								
Day 14–20	216^a^	0.00	188^b^	10.0	216^a^	0.00	192^b^	7.47
Day 21–27	216^a^	0.00	210^b^	3.83	216^a^	0.00	220^a^	10.0
Day 28–34	216^a^	0.00	198^b^	6.94	216^a^	0.00	211^a^	7.22
Day 35–41	216^a^	0.00	190^b^	8.69	216^a^	0.00	197^b^	13.6
Day 21–41	216^a^	0.00	198^b^	3.09	216^a^	0.00	208^a^	8.57
Ratio CD^CP+^/CD^CP−^-diet day 21–41	1.50^a^	0.00	1.01^b^	0.07	1.50^a^	0.00	1.28^a^	0.24
**DM-INTAKE [g/animal and day]**								
Day 14–20	81.7^ab^	4.72	76.4^b^	4.60	83.6^a^	2.13	79.2^ab^	4.35
Day 21–27	123^ab^	4.09	118^b^	4.22	125^a^	4.78	120^ab^	5.57
Day 28–34	162^ab^	5.89	156^b^	2.16	166^a^	7.61	157^b^	5.51
Day 35–41	180^a^	3.79	175^a^	8.65	177^a^	6.01	180^a^	5.93
Day 21–41	155^ab^	4.09	150^b^	4.12	156^a^	5.28	152^ab^	3.20
**CP-INTAKE [g/animal and day]**								
Day 14–20	17.7^a^	1.02	14.4^b^	1.38	18.1^a^	0.46	15.2^b^	0.68
Day 21–27	26.7^a^	0.89	24.8^b^	0.95	27.1^a^	1.03	26.3^a^	0.40
Day 28–34	34.9^ab^	1.27	30.9^c^	1.40	36.0^a^	1.65	33.2^ab^	1.15
Day 35–41	38.9^a^	0.82	33.2^b^	2.24	38.3^a^	1.30	35.3^b^	2.71
Day 21–41	33.5^a^	0.88	29.6^c^	0.73	33.8^a^	1.14	31.6^b^	0.76
AA_GL_ intake	9.36^a^	0.25	8.37^c^	0.21	9.43^a^	0.32	8.97^b^	0.25
AA_CM_ intake	13.2^a^	0.35	11.8^c^	0.29	13.3^a^	0.45	12.6^b^	0.30

*CN, without experimental C. jejuni infection; CP, with experimental C. jejuni infection; SP-diet, Standard protein diet; CD^CP+^-diet, High protein choice diet; CD^CP−^diet, Low protein choice diet; CD^CP+^/CD^CP−^diet, Choice diet; AA_GL_, Sum “growth limiting” amino acids like arginine, isoleucine, lysine, methionine, threonine and valine; AA_CM_, Sum of C. jejuni metabolisable amino acids like aspartic acid, glutamic acid, proline and serine*;

a,b *Values within a row with different superscripts differ significantly at p < 0.05*.

Between d 21 and d 42, the animals in the CD-diet groups ingested a diet with an average CP-content of 203 ± 8.00 g/ kg DM. This value was significantly lower than in the groups with SD-diets (*p* < 0.0001). Looking at the combined effects, it turned out that the CDCN group preferred, a bit more than common, the CD^CP−^-diet component. Therefore, birds consumed nearly identical proportions of both diets (relationship between CD^CP+^/CD^CP−^diets: 1.01 ± 0.07; *p* < 0.0001; to compare: theoretical CD^CP+^/CD^CP−^-ratio in the SDCN group was 1.50). This was fare away from the ratio three parts CD^CP+^-diet and two parts CD^CP−^-diet, if choice would have let to an identical botanical and chemical composition as found in the SD-diet. This shift in feed intake toward the protein-poor CD^CP−^-diet led to a significant reduction in average protein content of the ingested CD-diet. At 198 ± 3.09 g CP/ kg DM, the recorded crude protein content was significantly lower in the CDCN group (*p* = 0.0002) than in the SDCN group (216 g CP/ kg DM). Compared to the SDCP group (216 g CP/ kg DM), the animals in the experimental group infected with *C. jejuni* (CDCP) showed no significant difference concerning the crude protein content in the ingested feed (208 ± 8.57 g CP/ kg DM).

The differentiation at weekly level showed that in the period prior to the experimental infection (d 14–d 20), offering the choice diets led to ingestion of a diet with a significantly lower protein content (SD-diet: 216 g CP/ kg DM; CD-diet: 190 ± 8.68 g CP/kg DM; *p* < 0.0001). Comparing both choice diet groups (CDCN and CDCP), these groups had both a significantly lower (*p* = 0.0032 and *p* = 0.0020) crude protein density in diets (188 ± 10.0 g CP/ kg DM and 192 ± 7.47 g CP/ kg DM, respectively) in comparison to the feeding groups offered the SD-diet (216 g CP/ kg DM).

**Figure 2:**
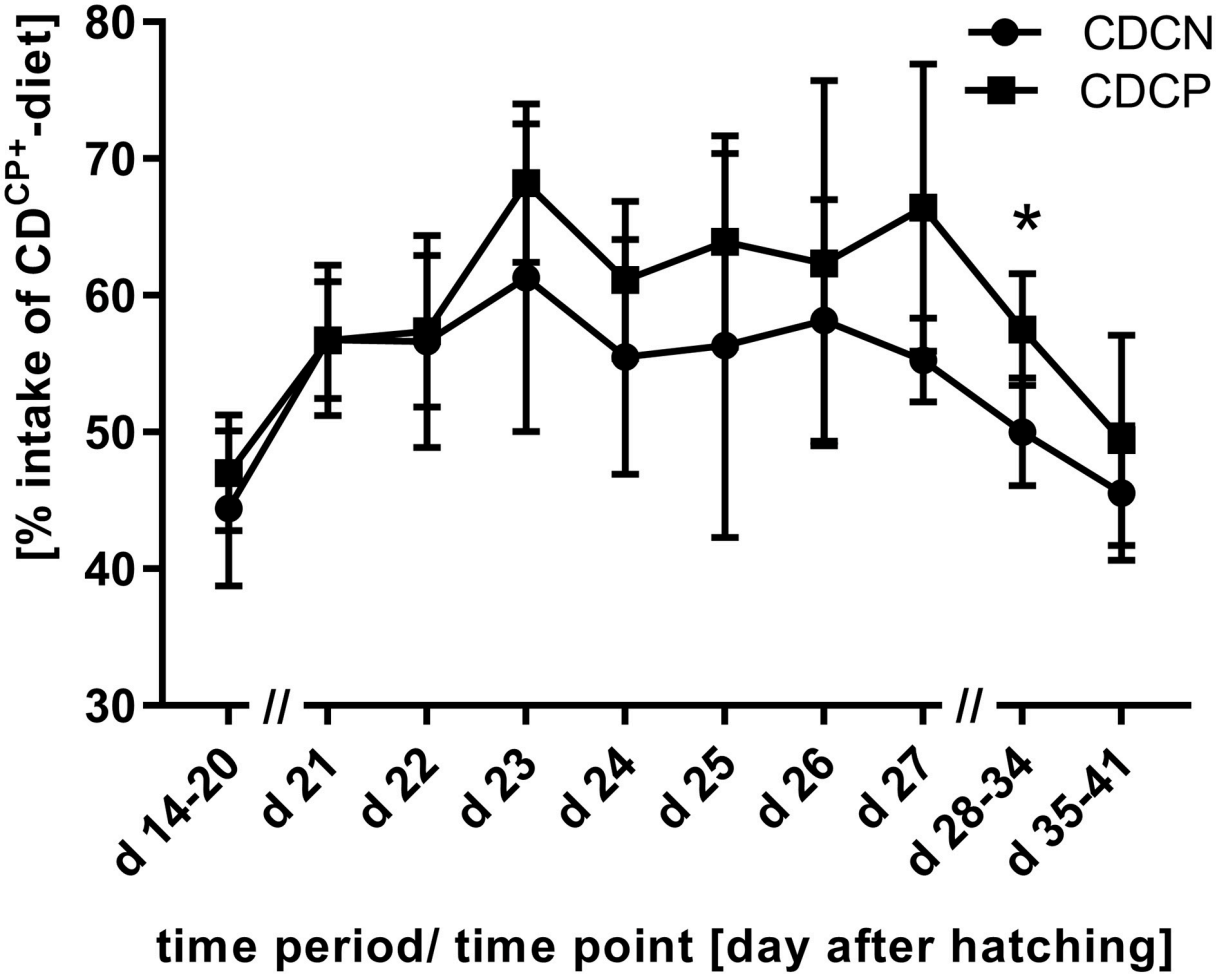
Proportion of uptake of the compound feed with high protein content (CD^CP+^-diet) in the experimental phase (experimental *C. jejuni* infection at the beginning of d 21; 60% intake of CD^CP+^-diet would mean a comparable diet composition to SD-diet); *Values differ significantly at *p* < 0.05.

In the first week after experimental infection (d 21–d 27), a numerically increased uptake of the high-protein CD^CP+^-diet was observed in both groups with choice option. Therefore, there was no difference depending the factor diet on weekly basis (SD-diet: 216 g CP/ kg DM; CD-diet: 215 ± 8.89 g CP/ kg DM; *p* = 0.5970; Table 5, Figure 2). For combined factors, the crude protein content of the ingested feed of the non-experimentally infected experimental group (CDCN) was lower (210 ± 3.83 g CP/kg DM; *p* = 0.0188) than in the SDCN group. The experimentally infected group (CDCP), on the other hand, chose a diet with a numerically higher crude protein content than in the infected group offered the SD-diet (220 ± 10.0 g CP/kg DM vs. 216 g/kg DM). In the two subsequent weeks a decline in intake of the protein-rich CD^CP+^-diet was seen in both experimental groups with choice (d 28–d 34, SD-diet: 216 g CP/kg DM; CD-diet: 204 ± 9.65 g CP/ kg DM; *p* = 0.0020; d 35–d 41, SD-diet: 216 g CP/kg DM; CD-diet: 193 ± 11.3 g CP/ kg DM; *p* < 0.0001). In the period from d 28–d 34, the share of intake of the CD^CP+^-diet was significantly lower in the CDCN group than in CDCP (Figure 2). Additionally, in both groups with choice diets, in the last week of the experiment the crude protein uptake was lower than in the groups with the SD-diet (CDCN: 190 ± 8.89; CDCP: 197 ± 13.6 g CP/ kg DM).

The SD-diet fed subgroups (SDCN, SDCP) showed a significantly higher daily feed intake per animal (156 ± 4.50 g) than the subgroups fed the CD-diet (151 ± 3.70 g) in the period from d 21 to d 42 (*p* = 0.0198). In combination with lower crude protein content of the CD-diets in the CDCN and CDCP subgroups, this resulted in a significantly (*p* < 0.0001) lower daily crude protein intake of the subgroups fed the CD-diets (30.6 ± 1.25 g) compared to the subgroups fed the SD-diets (33.7 ± 0.97 g). Week-by-week observation, however, showed that in the first week after infection, crude protein intake was lower in the CDCN group than in any other group. The CDCP group did not differ at this stage, nor in the subsequent week (d 28–34) from the groups which were offered the SD-diet.

The animals were randomly divided into four groups during the rearing phase, each housed in one box. At the end of the rearing phase, animals from each group were allocated to their respective five subgroups. Thus, there was no mixing between the animals of the groups. At the first body weight assessment (d 7), there was no difference in average body weight between the groups (Table 6). At d 14, broilers from the CDCN group had an average body weight lower than that in the other groups (average deviation: maximum 12 g/animal). However, at diet level (SD-diets and CD-diets), no statistical differences in the body weight of the animals were seen before the start of the experiment at d 14 (SD-diet: 494 ± 47.2 g, CD-diet: 488 ± 49.0 g). After the adaptation phase to the diet (d 14–d 20) and before experimental infection, the body weight of the SP-diet fed animals (987 ± 96.9 g) was higher than the body weight of CD-diet fed birds (908 ± 106 g; *p* < 0.0001). This difference was able to prevail during the experimental phase. At the day of dissection, the body weight of the feeding subgroups with SD-diets (3504 ± 428 g) was significantly higher than the body weight of the feeding subgroups with CD-diets (3,338 ± 465 g; *p* = 0.0013). The dressing percentage was also significantly more favorable in subgroups fed with the SD-diets (83.0 ± 1.18%) than in subgroups fed with the CD-diets (82.3 ± 1.21%; *p* < 0.0001).

**Table 6:** Performance data of broilers depending on experimental infection with *C. jejuni* and diet as well as feed choice.

**Item**	**CN**				**CP**			
	**SP-diet**		**CD**^**CP+**^**/CD**^**CP-**^**-diet**		**SP-diet**		**CD**^**CP+**^**/CD**^**CP-**^**-diet**	
	**Mean**	**SD**	**Mean**	**SD**	**Mean**	**SD**	**Mean**	**SD**
**BODY WEIGHT [in g]**								
Day 7	178^a^	20.3	175^a^	23.7	181^a^	19.9	181^a^	18.8
Day 14	491^ab^	46.0	479^b^	50.0	497^a^	49.0	497^a^	47.0
Day 21	984^a^	97.8	896^b^	105	990^a^	96.5	919^b^	108
Day 42	3273^a^	369	3037^b^	397	3307^a^	391	3242^a^	419
Dissection	3511^a^	436	3262^b^	456	3496^a^	423	3416^a^	465
Carcass weight^1^	2917^a^	359	2683^b^	380	2898^a^	350	2813^a^	388
Dressing %-age	83.1^a^	1.10	82.2^b^	1.39	82.9^a^	1.25	82.3^b^	0.984
WFR (g/g)	1.67^ab^	0.02	1.61^b^	0.06	1.68^a^	0.05	1.66^ab^	0.06
FCR (g/g)	1.61^b^	0.04	1.66^a^	0.03	1.60^b^	0.03	1.56^b^	0.05
CPE (kg/kg DM)	3.25^b^	0.08	3.44^a^	0.09	3.27^b^	0.06	3.49^a^	0.04

CN, without experimental C. jejuni infection; CP, with experimental C. jejuni infection; SP-diet, Standard protein diet; CD^CP+^-diet, High protein choice diet; CD^CP−^diet, Low protein choice diet; CD^CP+^/CD^CP−^diet, Choice diet; WFR, water-feed ratio in kg water intake / kg diet intake as fed; FCR, feed conversion ratio in kg diet intake as fed / kg body weight gain between Days 21–42; CPE, crude protein efficiency in kg body weight gain / kg crude protein intake;

1 *after exsanguation, evisceration and without head and legs, incl. feathers*;

a,b *Values within a row with different superscripts differ significantly at p < 0.05*.

Rating the body weight as a function of the main factor “infection” with *C. jejuni*, there were comparable starting conditions of non-infected and infected subgroups before the experimental infection at d 21 (940 ± 110 and 955 ± 108 g, respectively). At the end of the experiment, a significantly lower body weight of the non-infected subgroups (3,155 ± 400 g) in comparison to infected subgroups (3,275 ± 405 g; *p* = 0.0150) could be observed.

### Histology of the intestine

This histological examination showed a significantly deeper crypt depth in experimentally infected subgroups compared to the non-infected subgroups (256 ± 71.6 vs. 234 ± 61.3 μm; *p* = 0.0011). Regarding the main factor “diet” there were no significant differences. The feeding concept itself, however, had no effect on crypt depth (Table 7). The number of goblet cells was significantly higher in the CDCP subgroups.

**Table 7:** Crypt depth and the number of goblet cells in the caeca of broiler chickens.

**Item**	**CN**				**CP**			
	**SP-diet**		**CD**^**CP+**^**/CD**^**CP-**^**-diet**		**SP-diet**		**CD**^**CP+**^**/CD**^**CP-**^**-diet**	
	**Mean**	**SD**	**Mean**	**SD**	**Mean**	**SD**	**Mean**	**SD**
Crypt depth [μm; *n* = 25 per group]	235^b^	66.1	233^b^	56.4	263^a^	65.7	249^ab^	76.6
Goblet cells per standard crypt^a^ [*n* = 25 per group]	15.6^b^	5.88	15.4^b^	6.01	15.6^b^	5.48	17.2^a^	6.30

CN, without experimental C. jejuni infection; CP, with experimental C. jejuni infection; SP-diet, Standard protein diet; CD^CP+^-diet, High protein choice diet; CD^CP−^diet, Low protein choice diet; CD^CP+^/CD^CP−^diet, Choice diet; ^1^Determined goblet cell number to 250 μm crypt depth;

a,b *Values within a row with different superscripts differ significantly at p < 0.05*.

### Mucins in excreta

The total mucin content in the excreta showed a continuous increase from d 21 to d 42. An exception was the CDCN group (Table 8). In this group, the mucin levels slightly declined.

**Table 8:** Total mucin content of the excreta of broiler chickens.

**Mucin content [g/kg DM; *n* = 15 per group and time point]**	**CN**				**CP**			
	**SP-diet**		**CD**^**CP+**^**/CD**^**CP-**^**-diet**		**SP-diet**		**CD**^**CP+**^**/CD**^**CP-**^**-diet**	
	**Mean**	**SD**	**Mean**	**SD**	**Mean**	**SD**	**Mean**	**SD**
Day 21	56.3^ab^	4.82	57.4^a^	4.62	54.1^ab^	6.11	53.2^b^	5.01
Day 42	62.9^ab^	6.30	56.4^c^	4.18	64.5^a^	4.12	60.7^b^	4.38

CN, without experimental C. jejuni infection; CP, with experimental C. jejuni infection; SP-diet, Standard protein diet; CD^CP+^-diet, High protein choice diet; CD^CP−^diet, Low protein choice diet; CD^CP+^/CD^CP−^diet, Choice diet;

a,b *Values within a row with different superscripts differ significantly at p < 0.05*.

There was no difference in the total mucin content between the feeding concepts at d 21 (SD-diet: 55.2 ± 5.52 g/kg DM; CD-diet: 55.3 ± 5.19 g/kg DM). At d 42, however, a significantly higher total mucin content of groups fed the SD-diet (63.7 ± 5.30 g/kg DM) in comparison to groups fed the CD-diet was observed (58.5 ± 4.74 g/kg DM; *p* = 0.0002).

At d 21, the excreta of birds in groups which were later not experimentally infected, contained a significantly higher (56.8 ± 4.67) total mucin content compared to those experimentally infected with *C. jejuni* (53.7 ± 5.51 g/kg DM; *p* = 0.0188) at a later stage. By contrast, at d 42, the total mucin content of the *C. jejuni* infected groups was significantly higher (62.6 ± 4.62 g/kg DM) than in the non-infected groups (59.6 ± 6.21 g/ kg DM; *p* = 0.0396).

## Discussion

In a 2 × 2 factorial design, the effects of an experimental *C. jejuni* infection (without/with) on feed intake preference were analyzed in 300 broiler chickens allocated to 20 subgroups. Simultaneously, the course of this experimental infection, the effects on performance, the number of goblet cells in the ceca, and the concentration of mucins in excreta were analyzed. For this purpose, a choice diet set-up was established in broilers. The birds received either a standard diet with moderate protein content or a choice diet consisting of two supplement diets with low or high protein content.

### *campylobacter* excretion

Overall, there were only small differences in the excretion and spread of *C. jejuni* between the feeding concepts. The number of positive animals at day 22 in the CDCP group tended to be slightly higher (*p* = 0.1162). Overall, the CD^CP+^-component in the CD-diet had the highest proportion, with 68% at day 23. The crude protein uptake between the two experimentally infected groups did not differ significantly during the 21-day experimental phase. A significant impact on the dynamics of infection was therefore not expected. At dissection, counts of *C. jejuni* in cecal content were nearly identical between groups (7.02 ± 0.85/6.87 ± 1.12 log_10_ CFU *C. jejuni* per g (gram) of content in SDCP or rather CDCP birds). The absolute values are in line with data from Humphrey et al. (37), who found *C. jejuni* within the same range in cecal content after experimental infection at a dosage of about 2 × 10^5^ CFU per bird. Therefore, the experiments are generally well suited to test the effects of the infection on the behavioral choices of the animals with respect to the feed.

### Feed intake and performance

In both experimental groups with choice diets, in the period before the experimental infection, the feeding behavior was nearly identical. In the period after the experimental infection, the protein intake in the experimentally infected *C. jejuni* group was significantly higher between d 21–27 and d 28–34 than in the group without infection. So far, there are no experiments that have tested this effect in broiler chickens. From the experiments carried out by Han et al. (38) one can deduce that at a higher protein content in the diet (broiler feed vs. laying hen feed), the animals showed a higher *C. jejuni* load. In sheep lambs, Kyriazakis et al. (39) analyzed the intake of feed of different crude protein contents for subclinical nematode infections. Compared to negative control animals, lower feed intake was observed in the infected animals, with high protein components being ingested in higher proportions.

The question is what leads to the altered feed intake—the immunological response of the animal to the pathogen and the corresponding nutritional requirements in order to defend the pathogen, or the interaction between pathogens and feed intake regulation mechanisms.

A formation of immunologically important protein compounds and gluconeogenesis from metabolites of amino acid degradation cause an increased energy and nutrient consumption (18, 20, 40). The effect of immune stimulation on feed intake behavior is dependent on the duration of this stimulus (41). Stimulation of the immune system observed in association with *C. jejuni* infections is usually short (42). Rather, after an initial response of the immune system to the pathogen, the cytokine level associated with tolerance of the pathogen by the broiler immune system decreases (42, 43). One has to discriminate between nutrition and the kind of infection. For lysine, antiviral effects are known (44, 45). Collectively, arginine supplementation attenuated the overexpression of pro-inflammatory cytokines in lipopolysaccharide-induced inflammatory response probably through the suppression of the TLR4 pathway and CD14+ cell percentage (46). Furthermore, excessive arginine supplementation (1.76%) suppressed the percentages of circulating and splenic B cells (46). Expression of both TLR4 and TLR21, but not TLR2, is readily increased (6 h post infection) in cecal tissues in response to *C. jejuni* inoculation in 1-day-old birds, whereas in 2- and 4-week-old broiler chicks this is accompanied, however, by only a limited cytokine gene expression (43). In our study, the differences in the individual amino acid contents were attributed to the different amino acid patterns of wheat and soybean meal, which were used at different levels in the two compound feeds of choice. In the CD^CP+^-diet, there was 19.7 g/kg DM of arginine, whereas in the CD^CP−^-diet, we had only 4.72 g/kg DM of this amino acid (factor 4.17). However, due to the age of infection (d 21), it seems unlikely that an increased need for pathogen defense primarily led to an altered amino acid uptake.

Referring to a hypothesized interaction between pathogens and feed intake regulation mechanisms, aspartic acid intake could be interesting. The greatest difference between the CD^CP+^-diet and the CD^CP−^-diet was found in the content of aspartic acid (factor 5.23). This amino acid is preferred by *C. jejuni* for its growth (4, 13). Accordingly, the pathogen may also benefit from a high intake of the CD^CP+^-diet. Microbes may do this by way of two potential strategies: First, by generating cravings for feeds that they specialize on, or feeds that suppress their competitors. Second, by inducing dysphoria until the organism eats foods that enhance its fitness (47). The early pattern of brain activation following *C. jejuni* infection is characteristic of visceral sensory challenges, which modulate digestion and ingestive behavior (48). The strain used in this study was not further described in terms of its potential to generate an immunological response that could have altered feed intake behavior. Clinically, however, no evidence of infection-related changes in the animals were visible.

Furthermore, according to Awad et al. (49), the infection with the pathogen induces intestinal histomorphological changes, most prominently including a decrease in villus height, crypt depth and villus surface. Therefore, there is every indication that *Campylobacter* can, indeed, alter absorptive surface area with indirect negative consequences for production efficiency (49). No reduction in performance was found in the present study. In principle, however, a poorer absorption would also explain a preference for the component with a higher proportion of essential amino acids.

The growth performance of the animals in the experimental group (CDCP) during the experimental period of 21 days was comparable to that of the animals in the SDCP and SPCN groups. The body weight gain during the experimental period (d 21–42) was significantly (*p* < 0.0001) greater than the target of the Aviagen Group (1,880 g) in all groups. Also, the body weight on d 42 was significantly (*p* < 0.0001) higher than the target of 2,809 g (50).

### Histology of the intestine

The caeca are the preferred colonization sites in the digestive tract of broiler chickens (51). In the present study, the crypts in the cecum of infected animals receiving a standard diet were significantly deeper than in non-infected animals. Infected animals receiving CD-diets showed no difference in crypt depth from all other groups, but showed a significantly increased number of goblet cells. These results are in line with those of Beery et al. (51) who found significantly deeper cecal crypts in animals infected with *C. jejuni*. In particular, in the mucous layer of these crypts, a high colonization density by *C. jejuni* is observed (51, 52). Deeper crypts mean a larger surface of the cecal mucosa and thus a larger surface of the intestinal mucous layer. This could potentially benefit *C. jejuni*.

### Mucins in the excreta

In this study, experimental infection with *C. jejuni* had a positive effect on mucin secretion. This increased regardless of the type of feed supply. In the literature, altered secretion of certain mucins is described by the presence of *C. jejuni* (53, 54). (53) were able to demonstrate in the mouse model that infection with *C. jejuni* caused a specific mucin (MUC1) to be released more frequently. Stimulation of the corresponding gene by the presence of *C. jejuni* was also observed in humans (54). This membrane-bound mucin has an important function in defending the host organism against invasion of the pathogen and the release can therefore be regarded as a physiological response of the host organism (53, 55). Furthermore, generally an increase in mucin release is described by inflammatory changes in the intestinal mucosa (56–58). A stimulatory effect by *C. jejuni* on the immune system is known (49, 59–61). Thus, due to certain surface structures (pathogen-associated molecular patterns) of corresponding receptors (pattern recognition receptors) of the intestinal immune system of the broiler, *C. jejuni* is recognized as potentially pathogenic. Furthermore, it induces an increase in proinflammatory cytokines such as IL-1 and IL-6, which function as messenger substances in the organism (59–62). In addition to many other functions, these messenger substances also activate mucin release (56–58). Overall, there was no evidence of there being any effects of *C. jejuni* infection on performance and health. Therefore, the potential effects of inflammation are maximally local in nature.

## Conclusion

In this study, it could be proven that *C. jejuni* alters feed intake behavior toward higher protein intake. This had a positive effect on performance of the infected animals. Effects of infection on performance did not exist. Future studies should focus on potentially changing the diet composition away from the *C. jejuni-*induced pathway toward an increased protein uptake. For this dietetic concept for *Campylobacter* colonization, further studies are needed.

## Author contributions

CV was the initiators of the idea. CV and AH designed the study. LK, JH, RB, ML, and CV performed the study and made the analyses. LK and CV did the statistics. CV wrote the paper. All authors read and approved the final manuscript.

### Conflict of interest statement

AH is an employee of Evonik Nutrition & Care GmbH, Hanau-Wolfgang, Germany. The remaining authors declare that the research was conducted in the absence of any commercial or financial relationships that could be construed as a potential conflict of interest.
